# Sex-specific exposures and sex-combined outcomes in two-sample Mendelian randomization may mislead the causal inference

**DOI:** 10.1186/s13075-022-02922-7

**Published:** 2022-10-24

**Authors:** Zhenqian Wang, Jiawen Lu

**Affiliations:** School of Public Health (Shenzhen), Sun Yat-Sen University, Shenzhen, Guangdong China

**Keywords:** Two-sample Mendelian randomization, Sex-specific, Sex-combined, Samples

## Abstract

With great interest, we have read the recent article “Age at menarche, age at natural menopause, and risk of rheumatoid arthritis — a Mendelian randomization study” by Zhu et al. While we have a great appreciation for the work conducted by the authors, there are some methodological issues that need to be reconsidered. First, the gender description of the sample for age at first birth in this study is wrong according to the original genome-wide association study. Second, the study exploited sex-specific SNPs for age at menarche (AAM) and age at natural menopause (ANM) but sex-combined effects of the SNPs on rheumatoid arthritis (RA) that possibly lead no evidence for the causation of AAM and ANM on RA. We suggested the author add the possible biases due to the issue in the limitations. With problems mentioned above, we recommend solutions to make this article more perfect.

Dear Editor,

We read with great interest the paper by Zhu et al. [1], just published in the *Arthritis Research & Therapy*, where they provided a new genetic vision that no evidence supported the causal effect of reproductive factors on rheumatoid arthritis (RA). The authors used age at menarche (AAM), age at natural menopause (ANM) and age at first birth (AFB) to proxy hormonal reproductive characteristics and performed several Mendelian randomization (MR) methods with different assumptions about pleiotropy to robustly assess the causal effect of reproductive factors on RA. While we have a great appreciation for the work conducted by the authors, there are some methodological issues that need to be reconsidered.

First, the authors described they used a genome-wide association study (GWAS) summary-level data of female AFB (*N* = 251,151). However, Barban et al. conducted GWAS of both sexes including 251,151 individuals for AFB in the original paper [2]. By comparing the associations between SNPs and AFB mentioned in supplementary table 3 in this paper with table 1 in the original GWAS paper, we assume that the authors may misuse the sex-combined summary-level GWAS data of AFB.

Second, the authors listed the reasons why this MR study did not identify a putative causal link between three hormonal exposures and risk of RA despite the positive associations were pointed in conventional epidemiology studies [1]. However, there is another important methodological reason underlying such a discrepancy. The study exploited sex-specific SNPs for AAM and ANM but sex-combined effect of the SNPs on RA. An important assumption to ensure the validity of the two-sample MR study is that the two sets of samples should take from the same underlying population [3]. For example, the samples are similar in age and sex distribution and the same ethnic group. If this is not the case, then causal inferences may be inaccurate, as the association of the genetic variants with the outcome may not be replicated in the set of individuals in which the association with the exposure is estimated [4]. When the exposure of interest can only occur in males or females (e.g., prostate cancer, AAM, or ANM), ideally one would want the associations between SNPs and outcome estimate to be sex-specific. If that is not possible, it is important to draw on other external evidence to consider the extent for genetic architecture of outcome to be similar in females and males. In the two-sample MR study, the GWAS for AAM and ANM including only women, whereas GWAS of RA was assessed involved both men and women [5]. Moreover, RA is more prevalent in women and have the sex-specific genetic factors play an important role in RA susceptibility. For instance, sex differences in associations between the interferon-γ (*IFNG*) locus and RA in women only [6], whereas the leukocyte immunoglobulin-like receptor A3 (*LILRA3*) variant has shown increased association with RA, especially in men [7]. Therefore, they might not identify the causal effects of AAM and ANM on RA for the methodological issue. We suggested the author to add the possible biases due to the issue in the limitations.

## Data Availability

Not applicable.

## Abbreviations

AAM: Age at menarche
AFB: Age at first birth
ANM: Age at natural menopause
GWAS: Genome-wide association study
IFNG: Interferon-γ
LILRA3: Immunoglobulin-like receptor A3
MR: Mendelian randomization
RA: Rheumatoid arthritis
